# Role of Editorial and Peer Review Processes in Publication Bias: Analysis of Drug Trials Submitted to Eight Medical Journals

**DOI:** 10.1371/journal.pone.0104846

**Published:** 2014-08-12

**Authors:** Marlies van Lent, John Overbeke, Henk Jan Out

**Affiliations:** 1 Clinical Research Centre Nijmegen, Department of Pharmacology – Toxicology, Radboud University Medical Centre, Nijmegen, The Netherlands; 2 Department of Primary and Community Care, Radboud University Medical Centre, Nijmegen, The Netherlands; 3 Teva Pharmaceuticals, Amsterdam, The Netherlands; Johns Hopkins Bloomberg School of Public Health, United States of America

## Abstract

**Background:**

Publication bias is generally ascribed to authors and sponsors failing to submit studies with negative results, but may also occur after submission. We evaluated whether submitted manuscripts on randomized controlled trials (RCTs) with drugs are more likely to be accepted if they report positive results.

**Methods:**

Manuscripts submitted from January 2010 through April 2012 to one general medical journal (*BMJ*) and seven specialty journals (*Annals of the Rheumatic Diseases*, *British Journal of Ophthalmology*, *Gut*, *Heart*, *Thorax*, *Diabetologia*, and *Journal of Hepatology*) were included, if at least one study arm assessed the efficacy or safety of a drug and a statistical test was used to evaluate treatment effects. Publication status was retrospectively retrieved from submission systems or provided by journals. Sponsorship and trial results were extracted from manuscripts and classified according to predefined criteria. Main outcome measure was acceptance for publication.

**Results:**

Of 15,972 manuscripts submitted, 472 (3.0%) were drug RCTs, of which 98 (20.8%) were published. Among submitted drug RCTs, 287 (60.8%) had positive and 185 (39.2%) negative results. Of these, 60 (20.9%) and 38 (20.5%), respectively, were published. Manuscripts on non-industry trials (n = 213) reported positive results in 138 (64.8%) manuscripts, compared to 71 (47.7%) on industry-supported trials (n = 149), and 78 (70.9%) on industry-sponsored trials (n = 110). Twenty-seven (12.7%) non-industry trials were published, compared to 27 (18.1%) industry-supported and 44 (40.0%) industry-sponsored trials. After adjustment for other trial characteristics, manuscripts reporting positive results were not more likely to be published (OR, 1.00; 95% CI, 0.61 to 1.66). Submission to specialty journals, sample size, multicentre status, journal impact factor, and corresponding authors from Europe or US were significantly associated with publication.

**Conclusions:**

For the selected journals, there was no tendency to preferably publish manuscripts on drug RCTs that reported positive results, suggesting that publication bias may occur mainly prior to submission.

## Introduction

Publication bias refers to the selective publication of research findings depending on the nature and direction of results [1] and has been widely studied. Studies reporting positive results are more likely to be published [2]–[4], which may cause meta-analyses based on published reports to overestimate the size of apparent treatment effects. Pharmaceutical industry sponsorship has particularly been associated with publication of favourable outcomes.[5]–[8] Publication bias is generally ascribed to authors and sponsors failing to submit studies with negative results, but may also occur once manuscripts have been submitted to journals.[9], [10]

A limited number of studies have systematically evaluated publication bias in editorial decision making. Olson et al. assessed manuscripts submitted to JAMA, and found no difference in publication rates between manuscripts with positive versus negative results.[11] Lee et al. found similar results for manuscripts submitted to BMJ, the Lancet and Annals of Internal Medicine.[12] Lynch et al. and Okike et al. assessed submissions to The Journal of Bone and Joint Surgery, and found no evidence for publication bias by editors.[13], [14] Overall, these studies suggest that submitted manuscripts with positive results are not more likely to be published, which was confirmed by a recent meta-analysis.[15]

However, these studies had certain limitations. Most were prospective studies, so editors and reviewers may have been aware that some investigation was in progress.[11]–[13] This possibly influenced their decision making, even if they were not informed about the study hypothesis. Olson et al. and Lee et al. included large general medical journals with high impact factors, and their results may not be generalizable to specialty journals or journals with fewer submissions, fewer editors or lower circulation.[11] Two studies were limited to orthopaedic journals, and resulting findings may not apply to other specialties.[13], [14] Moreover, publication bias may affect studies with various designs and interventions differently. Olson et al. included manuscripts on controlled trials, while others enrolled manuscripts reporting original research, regardless of study design.[12]–[14] None of the studies that followed manuscripts submitted to journals included papers based on the intervention tested, while publication bias has predominantly been researched and described for drug trials.[4], [6], [7], [16], [17]

Acceptance rates may also depend on sponsorship, next to study results. Publication of industry-sponsored trials has been associated with an increase in journal impact factors [18], as impact factors depend on citation rates and industry-sponsored trials are more frequently cited than non-profit trials.[19], [20] Moreover, journals create revenue through reprint sales, and industry funding of trials has been associated with high numbers of reprint orders.[21], [22] Lynch et al. found that commercially funded research was more likely to be published, while Olson et al. reported no difference according to funding source.[11], [13] However, neither of these studies focused on drug research, in which industry funding appears to be most abundant.

In this study, we retrospectively assessed manuscripts on randomized controlled trials (RCTs) with drugs submitted to one general medical journal and seven specialty journals, and evaluated acceptance rates of manuscripts reporting positive versus negative results. We hypothesized that negative trials were less likely to be published. Submission rates of positive versus negative studies were compared by sponsor type and the influence of sponsorship on acceptance rates was determined.

## Methods

### Selection of journals

Editors of six major general medical journals were asked for their cooperation to provide access to submitted manuscripts, peer review comments, and final decisions on publication. BMJ agreed to participate and the BMJ Group also provided access to data of BMJ specialty journals. In addition, other European specialty journals were asked to participate. All journals were selected based on 1. impact factor (journals indexed with the highest impact factors within subject categories, according to the Institute for Scientific Information Journal Citation Report 2011); and 2. the number of drug RCTs published in 2010–2011, determined on the basis of a PubMed search. As a result, publication outcomes were studied for one general medical journal and seven specialty journals: BMJ, Annals of the Rheumatic Diseases, British Journal of Ophthalmology, Gut, Heart, Thorax (all from the BMJ Group), Diabetologia, and Journal of Hepatology.

### Selection of submitted manuscripts

Original research manuscripts submitted between January 1, 2010 and April 30, 2012 were screened for eligibility by one author. The study time frame per journal was based on the retrospective period for which all required data, regardless of the publication status of manuscripts, was completely available in manuscript submission systems at the time of data extraction. Manuscripts reporting results of RCTs were selected, if at least one study arm assessed the efficacy or safety of a drug intervention (including vaccines, biologics, dietary supplements, and herbal medicinal products) and a statistical test was used to evaluate treatment effects. Post-hoc and subgroup analyses and follow-up studies of drug RCTs were included.

### Data extraction

Data were extracted retrospectively by one author using a standardized data extraction form. Primary outcome was acceptance for publication. Publication status and peer review details were retrieved from submission systems or provided by journals. Manuscripts were assessed as outright rejected, rejected after external peer review, or accepted for publication. Information on trial results and sponsorship was extracted from manuscripts. Data on study characteristics previously examined for association with publication (sample size, number of centres, corresponding author's country of residence [11]–[13]) were also retrieved. Manuscripts were searched for registration numbers to determine whether studies were registered in a trial registry that complies with requirements of the International Committee of Medical Journal Editors (ICMJE).[23] All included journals required trial registration in their instructions to authors.

### Classification of results and sponsorship

Trial results and sponsorship were classified based on consensus between two authors according to predefined criteria.[24] Briefly, outcomes were scored as positive if results reported for the primary endpoint were statistically significant (p<0.05 or 95% confidence interval [CI] for difference excluding 0 or 95% CI for ratio excluding 1) and supported the efficacy of the test drug, and negative if they did not. For equivalence and non-inferiority trials, results were classified as positive if treatments were equivalent. If the primary endpoint was a safety parameter, trials were classified as positive if the test drug was as safe as or safer than control. When explicitly hypothesized that the test drug was expected to be safer than control, results were categorized as negative if treatments were equally harmful. If no primary outcome was stated for a trial or multiple primary endpoints were selected, results were classified based on the statistical significance and direction of most (primary) outcomes (>50%). Studies were classified as non-industry, industry-supported or industry-sponsored trials. For non-industry trials, no associations with pharmaceutical companies were reported in the manuscript. Studies reporting donation of study medication or placebos by a manufacturer, studies stating receipt of financial support from a pharmaceutical company and studies with authors affiliated to industry were classified as industry-supported trials. For industry-sponsored trials, a pharmaceutical company was explicitly described as the study sponsor, or the company funding the trial was reported to have participated in the study design, data collection, analysis, preparation of the manuscript, and/or the decision to publish. When doubt remained over sponsorship, information in the trial registry took precedence over other information (if registered).

### Statistical analysis

The association between publication and trial results and other characteristics was first analyzed using univariate logistic regression. Associations between acceptance (versus rejection) and trial characteristics were estimated with odds ratios (ORs) and 95% CIs. P-values were not adjusted for multiple comparisons and P<.05 was considered statistically significant. To control for several characteristics simultaneously, multiple logistic regression was used and ORs were calculated. As 98 submitted manuscripts were accepted in this study, nine predictors could be entered in the model simultaneously, with ten acceptances per predictor. Besides the primary analysis (accepted vs all rejected manuscripts), two additional multivariable analyses were performed to compare accepted manuscripts with those outright rejected or rejected after peer review. These sensitivity analyses were conducted to assess whether the effects of the covariates were dependent on the type of rejection, i.e. whether the decision to reject manuscripts after initial editorial screening versus after peer review was of influence on the association between positive results and acceptance. Statistical analyses were performed using SPSS software (version 20; Chicago, Illinois).

### Ethics

To assure confidentiality of information in manuscripts and submission systems, the authors signed confidentiality agreements before gaining access to the data. As standard editorial processes were unchanged, authors and peer reviewers were not informed about this study. Approval from a research ethics committee was not required, as this study involved no human participants.

## Results

**Figure 1:**
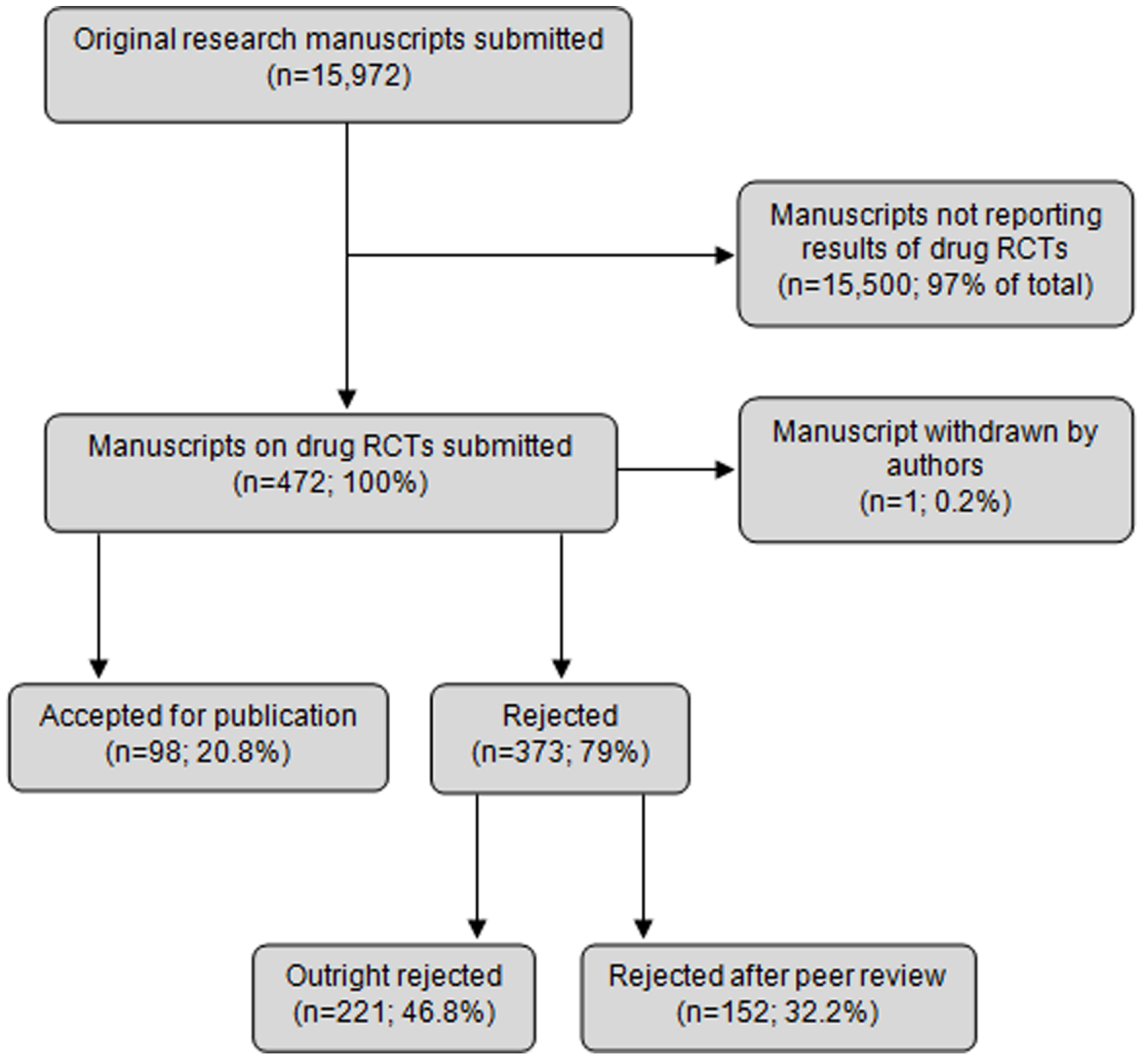
Publication status of manuscripts submitted to eight medical journals during the study time frame.

From January 2010 through April 2012, 15,972 manuscripts reporting original research were submitted to eight journals, of which 472 (3.0%) met all inclusion criteria. Ninety-eight manuscripts (20.8%) were published, 221 (46.8%) were outright rejected and 152 (32.2%) were rejected after peer review. One manuscript (0.2%) was withdrawn by authors before editorial decisions were made (Figure 1).

Among 472 drug RCTs, 287 (60.8%) had positive results and 185 (39.2%) had negative results (Table 1). Of these, 135 (47.0%) and 86 (46.5%), respectively, were rejected immediately, and 91 (31.7%) and 61 (33.0%) after peer review. In total, compared to the number of submitted manuscripts, 60 (20.9%) positive studies were published compared to 38 (20.5%) negative studies. Publication outcomes of manuscripts submitted to each individual journal are shown in Table 1. For all journals except Thorax, the proportion of submitted manuscripts with positive results outnumbered those with negative results. In the BMJ, British Journal of Ophthalmology, Diabetologia, Gut, Heart, and Journal of Hepatology, a higher proportion of submitted manuscripts with negative results were published, while in Annals of the Rheumatic Diseases and Thorax a higher proportion of positive studies were published.

**Table 1:** Publication status of submitted manuscripts reporting positive vs negative results by journal.

	Submitted		Submitted - Positive results				Submitted - Negative results			
	Positive (%)	Negative (%)	N	Outright rejected (%)	Rejected after review (%)	Published (%)	N	Outright rejected (%)	Rejected after review (%)	Published (%)
**Total manuscripts** (N = 472)*	287 (60.8)	185 (39.2)	287	135 (47.0)	91 (31.7)	60 (20.9)	185	86 (46.5)	61 (33.0)	38 (20.5)
**Journal**										
BMJ (N = 94)*	50 (53.2)	44 (46.8)	50	30 (60.0)	13 (26.0)	6 (12.0)	44	24 (54.5)	12 (27.3)	8 (18.2)
Ann Rheum Dis (N = 56)	37 (66.1)	19 (33.9)	37	4 (10.8)	8 (21.6)	25 (67.6)	19	6 (31.6)	5 (26.3)	8 (42.1)
Brit J Ophthalmol (N = 22)	15 (68.2)	7 (31.8)	15	8 (53.3)	5 (33.3)	2 (13.3)	7	1 (14.3)	4 (57.1)	2 (28.6)
Diabetologia (N = 135)	88 (65.2)	47 (34.8)	88	58 (65.9)	20 (22.7)	10 (11.4)	47	26 (55.3)	15 (31.9)	6 (12.8)
Gut (N = 61)	39 (63.9)	22 (36.1)	39	18 (46.2)	15 (38.5)	6 (15.4)	22	12 (54.5)	5 (22.7)	5 (22.7)
Heart (N = 24)	14 (58.3)	10 (41.7)	14	7 (50.0)	6 (42.9)	1 (7.1)	10	8 (80.0)	0 (0.0)	2 (20.0)
J Hepatol (N = 44)	28 (63.6)	16 (36.4)	28	5 (17.9)	16 (57.1)	7 (25.0)	16	1 (6.2)	9 (56.2)	6 (37.5)
Thorax (N = 36)	16 (44.4)	20 (55.6)	16	5 (31.2)	8 (50.0)	3 (18.8)	20	8 (40.0)	11 (55.0)	1 (5.0)

*1 manuscript with positive results submitted to the BMJ was withdrawn by authors before editorial decisions were made. N = number of submitted manuscripts.

Submitted manuscripts reporting non-industry trials (n = 213) had positive results in 138 manuscripts (64.8%), compared to 71 manuscripts (47.7%) on industry-supported trials (n = 149), and 78 manuscripts (70.9%) on industry-sponsored trials (n = 110) (Table 2). When all trials with industry involvement (n = 259) were taken together, 149 submitted manuscripts (57.5%) reported positive results. Twenty-seven (12.7%) non-industry trials were published, compared to 27 (18.1%) industry-supported trials, and 44 (40.0%) industry-sponsored trials.

**Table 2:** Publication status of submitted manuscripts reporting positive versus negative results by sponsor type.

	Submitted		Submitted - Positive results				Submitted - Negative results			
	Positive (%)	Negative (%)	N	Outright rejected (%)	Rejected after review (%)	Published (%)	N	Outright rejected (%)	Rejected after review (%)	Published (%)
**Total manuscripts** (N = 472)*	287 (60.8)	185 (39.2)	287	135 (47.0)	91 (31.7)	60 (20.9)	185	86 (46.5)	61 (33.0)	38 (20.5)
**Sponsorship**										
Non-industry (N = 213)	138 (64.8)	75 (35.2)	138	81 (58.7)	43 (31.2)	14 (10.1)	75	42 (56.0)	20 (26.7)	13 (17.3)
Industry-supported (N = 149)*	71 (47.7)	78 (52.3)	71	33 (46.5)	23 (32.4)	14 (19.7)	78	36 (46.2)	29 (37.2)	13 (16.7)
Industry-sponsored (N = 110)	78 (70.9)	32 (29.1)	78	21 (26.9)	25 (32.1)	32 (41.0)	32	8 (25.0)	12 (37.5)	12 (37.5)

*1 manuscript with positive results of an industry-supported trial was withdrawn by authors before editorial decisions were made. N = number of submitted manuscripts.

In the univariate analysis, manuscripts reporting positive results were not more likely to be published compared to those with negative results (OR, 1.03; 95% CI, 0.65–1.62) (Table 3). Sponsorship was significantly associated with publication; industry-sponsored trials were more likely to be published than non-industry trials (OR, 4.59; 95% CI 2.64–8.00). Trial registration, sample size, being a multicentre trial or follow-up study of an RCT, a corresponding author from Europe or the US, and the journal to which manuscripts are submitted were associated with the chance of publication (Table 3).

**Table 3:** Characteristics of submitted manuscripts and their association with publication: univariate analysis (accepted vs all rejected).

	Total number (%*)	Published number (%^§^)	Odds ratio (95% CI)	P-value
**Total manuscripts**	472 (100)	98 (20.8)		
**Results**				.909
Positive results	287 (60.8)	60 (20.9)	1.03 (0.65–1.62)	
Negative results	185 (39.2)	38 (20.5)	1.00	
**Journal (IF)**				.000
BMJ (14.093)	94 (19.9)	14 (14.9)	1.00	
Ann Rheum Dis (8.727)	56 (11.9)	33 (58.9)	8.10 (3.72–17.64)	
Brit J Ophthalmol (2.902)	22 (4.7)	4 (18.2)	1.25 (0.37–4.26)	
Diabetologia (6.814)	135 (28.6)	16 (11.9)	0.76 (0.35–1.64)	
Gut (10.111)	61 (12.9)	11 (18.0)	1.24 (0.52–2.95)	
Heart (4.223)	24 (5.1)	3 (12.5)	0.81 (0.21–3.07)	
J Hepatol (9.264)	44 (9.3)	13 (29.5)	2.37 (1.00–5.60)	
Thorax (6.840)	36 (7.6)	4 (11.1)	0.71 (0.22–2.31)	
**Journal type**				.130
General medical journal	94 (19.9)	14 (14.9)	0.62 (0.33–1.15)	
Specialty journal	378 (80.1)	84 (22.2)	1.00	
**Sponsorship**				.000
Industry-sponsored	110 (23.3)	44 (40.0)	4.59 (2.64–8.00)	
Industry-supported	149 (31.6)	27 (18.1)	1.54 (0.86–2.75)	
Non-industry	213 (45.1)	27 (12.7)	1.00	
**Industry involvement**				.000
Industry-supported or sponsored	259 (54.9)	71 (27.4)	2.62 (1.61–4.26)	
Non-industry	213 (45.1)	27 (12.7)	1.00	
**Trial registration**				.010
Yes	374 (79.2)	87 (23.3)	2.41 (1.23–4.71)	
No	98 (20.8)	11 (11.2)	1.00	
**Sample size**				.000
>100 participants	211 (44.7)	60 (28.4)	2.35 (1.49–3.70)	
≤100 participants	261 (55.3)	38 (14.6)	1.00	
**Number of centres**				.000
Multicentre	224 (47.5)	70 (31.2)	3.60 (2.22–5.84)	
Single centre	248 (52.5)	28 (11.3)	1.00	
**Study type**				.022
Posthoc/subgroup analysis RCT	72 (15.3)	15 (20.8)	1.11 (0.60–2.07)	
Follow-up study of RCT	19 (4.0)	9 (47.4)	3.73 (1.47–9.52)	
RCT	381 (80.7)	74 (19.4)	1.00	
**Authors' country of residence**				.003
Europe	224 (47.5)	57 (25.4)	2.42 (1.41–4.15)	
US	71 (15.0)	19 (26.8)	2.57 (1.29–5.13)	
Rest of the world	177 (37.5)	22 (12.4)	1.00	
**Journal impact factor**			1.02 (0.95–1.09)	.637

*Percentage of grand total of submitted manuscripts. ^§^ Percentage of row category that were accepted for publication. IF = journal impact factor, 2011.

In the multivariable analysis, accepted versus rejected manuscripts were compared after controlling for characteristics that were significantly associated with publication in the univariate analysis, or otherwise deemed important in relation to publication (Table 4). After adjustment for these variables, acceptance rates were not higher for trials with positive results than for trials with negative results (OR, 1.00; 95% CI, 0.61–1.66). The association of other factors with publication is shown in Table 4. In the multivariable analysis, industry-sponsorship and trial registration were no longer significantly associated with publication, while journal impact factor and submission to specialty journals were associated with an increased chance of acceptance. In the multivariable analyses comparing accepted manuscripts with those outright rejected or rejected after peer review, positive studies were not more likely to be published (Table 4). Findings of these analyses confirmed the primary analysis, as the direction of effects found was equal in all analyses. However, most associations were not statistically significant when comparing accepted manuscripts with those rejected after peer review.

**Table 4:** Characteristics of submitted manuscripts associated with publication: multivariable analysis.

	Accepted (n = 98) vs all rejected (n = 373)		Accepted (n = 98) vs outright rejected (n = 221)		Accepted (n = 98) vs rejected after review (n = 152)	
Characteristic	Odds ratio (95% CI)	P-value	Odds ratio (95% CI)	P-value	Odds ratio (95% CI)	P-value
Positive results vs negative	1.00 (0.61–1.66)	.994	0.94 (0.53–1.66)	.832	1.04 (0.59–1.84)	.881
BMJ vs specialty journals	0.16 (0.05–0.53)	.003	0.07 (0.02–0.28)	.000	0.40 (0.11–1.52)	.177
Industry-sponsored vs non-industry	1.79 (0.90–3.58)	.097	2.35 (1.06–5.23)	.036	1.41 (0.64–3.06)	.393
Industry-supported vs non-industry	1.13 (0.60–2.13)	.698	1.27 (0.64–2.51)	.489	0.94 (0.47–1.89)	.858
Trial registration vs not registered	1.55 (0.75–3.20)	.237	1.21 (0.55–2.68)	.640	1.86 (0.84–4.12)	.127
Number of participants >100 vs ≤100	1.91 (1.11–3.31)	.020	2.34 (1.25–4.37)	.008	1.47 (0.78–2.74)	.231
Multicentre vs single centre	1.87 (1.02–3.40)	.042	2.12 (1.07–4.18)	.030	1.65 (0.84–3.21)	.145
Journal impact factor	1.19 (1.02–1.39)	.030	1.29 (1.08–1.53)	.006	1.09 (0.93–1.29)	.300
Corresponding authors' country of residence EU or US vs rest of the world*	2.02 (1.14–3.57)	.015	1.77 (0.94–3.34)	.078	2.07 (1.12–3.84)	.020

*Collapsed categories: Europe and US vs rest of the world.

## Discussion

This is the first study that evaluated publication bias of manuscripts submitted to both a general medical journal and multiple specialty journals. Submitted manuscripts on drug RCTs were not more likely to be published if they reported positive results, regardless of whether rejected manuscripts were peer reviewed or not. This confirms findings of previous studies that followed manuscripts submitted to journals.[11]–[14] The proportion of submitted manuscripts with positive results outnumbered those with negative results, suggesting that publication bias mainly occurs prior to submission. This corresponds to findings of surveys among investigators on reasons for non-publication of results showing that studies primarily remained unpublished due to investigator-related factors.[25], [26]

Both submitted non-industry and industry-sponsored trials were more likely to report positive results, in contrast to study findings indicating that particularly industry sponsorship is associated with favourable outcomes.[5], [6] Interestingly, industry-sponsorship was associated with publication in the univariate analysis, as was previously found by Lynch et al.[13] This could be related to editorial decisions, as incentives such as citation rates [19], [20] and reprint revenue [21], [22] could favour the acceptance of these studies. Trial registration resulted in an increased unadjusted OR for publication, which may reflect that included journals adhere to ICMJE policy requiring registration as a condition of consideration for publication. Multicentre trials and studies enrolling more than 100 participants were more likely to be published, which was in agreement with findings of previous studies.[11], [12]

Previous studies found that manuscripts whose corresponding author was from the same country as the publishing journal were more likely to be accepted.[12], [13], [27] We included European journals only and found that having a corresponding author from either Europe or the US increased the chance of publication. This may result from a ‘familiarity effect’, leading reviewers and editors to be more accepting of trials with familiar interventions, clinical relevance, and language use.[13], [28]

After adjustment for other trial characteristics, submission to specialty journals was associated with publication. This seems plausible, as acceptance rates of general medical journals are known to be lower than those of specialty journals. A higher journal impact factor increased the chance of publication, though high impact journals generally have low acceptance rates. The direction of this association may be explained by relatively high acceptance rates found for two journals (Annals of the Rheumatic Diseases, Journal of Hepatology). Studies with negative results submitted to Annals of the Rheumatic Diseases and Thorax seemed less likely to be published. In view of the fact that BMJ was the only general medical journal that was included in this study and the number of accepted manuscripts per journal was relatively low, these data need to be interpreted with caution.

The retrospective design of this study overcomes limitations that prospective studies on publication bias in editorial decision making have. To study publication bias after manuscript submission, collaboration from editors is essential. In prospective studies, the decision-making behaviour of editors may be influenced by awareness of an ongoing investigation [11], introducing bias into the selection of manuscripts that are published. However, due to this retrospective design, our study time frame was limited by the retrospective availability of data in manuscripts submission systems.

We included a general medical journal and specialty journals across different medical specialties, which increases the generalizability of our results compared to studies that only included large general medical journals or an orthopaedic journal.[11]–[14] However, we acknowledge that the journals included in our study are merely a sample of all peer reviewed medical journals. It might be that those journals that agreed to participate, did so based on existing editorial policy to publish papers of scientific worth regardless of the direction of results. As both BMJ and 5 of the 7 included specialty journals are published by the BMJ Group, the results of this study may have been affected by clustering effects based on publisher policy. Furthermore, investigators may prefer to submit large, multi-centre, well-conducted studies to high impact journals like those included in our study. If publication bias is more likely to affect smaller studies, the inclusion of lower impact journals that more commonly receive smaller, single-center or negative studies might have influenced our results. However, no study has found evidence for publication bias in editorial decision making, irrespective of its design or included journals.

Other strengths include the objective selection criteria for journals and manuscripts, analysis of confounding characteristics, and classification of results and sponsorship based on predefined criteria.[24] Assessment of results and establishing the role of the funding source may appear to be straightforward, but in most studies on publication bias, methods for classification of results and sponsorship are only reported to a limited extent and definitions used are inconsistent across studies.[24]

This study has certain limitations. During the assessment of results and other characteristics, there was no blinding for publication status. In a retrospective study, blinding for publication status would require editors to redact information made available to investigators, which could introduce substantial bias. Furthermore, the screening and selection of manuscripts and the extraction of manuscript characteristics were performed by one author, while this would ideally have been done by two independent assessors. We have focused on drug RCTs, and our results may not be generalizable to studies with different designs or interventions. The number of submitted drug RCTs varied between journals. This could be related to medical specialty and journal impact factor, but may also vary due to differences in retrospective availability of data in submission systems of journals. However, the proportion of drug RCTs among submitted manuscripts was comparable for all journals. We included European journals only, and editorial processes might slightly differ compared to US journals. Our study included a representative sample of drug RCTs though, as more than half of all submitted trials were from outside Europe.

In this study, we evaluated the overall editorial process after manuscript submission and have not specifically examined the role of peer reviewers in publication bias. Abbot and Ernst tested whether publication bias was present during peer review, and found that reviewers were no more likely to recommend publication of a fictitious manuscript with positive results.[29] However, Emerson et al. showed that a fabricated manuscript reporting positive results was more often recommended for publication than an otherwise identical manuscript reporting no effect.[10] It is difficult to assess the extent to which editors' decisions were reliant on reviewers' comments in this study. Kravitz et al. found that editors tend to place considerable weight on reviewers' recommendations.[30]

Finally, we have not determined quality scores for included trials. Lee et al. found an increased chance of acceptance for manuscripts with high quality scores.[12] The fact that multicentre and large (>100 participants) trials were more likely to be published can be seen as a proxy for quality. However, Lynch et al. found no relation between quality scores and publication.[13] Though observed acceptance rates did not favour manuscripts with positive results in our study, negative studies may have been of higher quality than positive studies, as was found by Lynch et al.[13] This could result from authors believing that negative papers are less likely to be accepted, therefore only submitting those of high quality. As a consequence, submitted negative manuscripts may be of higher quality than positive manuscripts. Editorial bias occurs if submitted negative studies, although superior in quality, are not more likely to be published.[31] However, we found no differences between positive and negative manuscripts regarding sample size and multicentre status.

To reduce potential publication bias after submission, editors and peer reviewers could be blinded to results and discussion sections of manuscripts.[9], [32], [33] Preliminary decisions would be based on review of introduction and methods sections, and if manuscripts pass this initial stage, the full article could be provided to make a final evaluation. An RCT in which submitted manuscripts are randomized to either traditional review or review with initial blinding to results could confirm whether editors are not more likely to accept positive studies. However, no journals have implemented this two-stage review so far.

In conclusion, we found that for the sample of selected journals, there was no tendency to preferably publish submitted manuscripts on drug RCTs that reported positive results. The proportion of submitted manuscripts with positive results outnumbered those with negative results irrespective of sponsor type, suggesting that publication bias may occur mainly before manuscripts are submitted to journals.
